# Insect Life

**Published:** 1846-04

**Authors:** 


Art. II.—*Insect Life.* By David Badham, m.d., late Radcliffe Travelling Fellow of the University of Oxford, &c.—*London,* 1845. 12mo, pp. 171.

This little work is nothing else than a new edition of a pamphlet which was published some years since under the title of 'The Question concerning the Sensibility, Intelligence, and Instinctive Actions of Insects,' and which we noticed in our Fifth Volume, (p. 543.) Its object is to prove the startling proposition that *insects have no sensation;* but we do not perceive that the author has advanced one whit further in his argument than he did before. The following are the assumptions on which it is founded: 1. Sensation is inconceivable as existing apart from intelligence and spontaneous movement. 2. The actions of insects and the organization of their nervous system, indicate that they do not possess intelligence. 3. Therefore, as sensation cannot exist without intelligence, insects have no sensation.

Now we may admit with the author that the greater part of the actions of insects, if not all, are executed without the exercise of any intelligence, or designed adaptation of means to ends, on their own parts, and yet contest the position that sensation cannot exist, and give rise to muscular movements, without intelligence and voluntary power. What, for example, is more involuntary than the act of vomiting? yet this results from sensations with which the intelligence has nothing to do,—as when provoked by an offensive odour, the sight of a disgusting object, the tickling of the fauces with a feather, &c. This is only one of a large group of *consensual* acts, which we regard as having a character distinct from the reflex on the one hand, in that they require sensation as a link in the chain of their performance; and as equally distinct from those prompted by intelligence, in being performed independently of the will, and even in opposition to it.

The fundamental positions taken up by the author being so palpably erroneous, (in our estimation, at least,) we do not think it necessary to enter into a detailed examination of his arguments, or of his supposed facts. We think that we could point out a considerable number of inconsistencies and non-sequiturs in the former; and as to the latter we could almost undertake to show a blunder in every page. We think that the Radcliffe Travelling Fellow cannot have derived much advantage from his appointment, if he can produce no better fruits of it than his "Insect Life."

